# Clinical and financial significance of insomnia within a large payor-provider health system

**DOI:** 10.1093/sleepadvances/zpae054

**Published:** 2024-07-27

**Authors:** Bradley E Karlin, Ryan J Anderson, Jillian M Rung, Charlotte Drury-Gworek, Tyson S Barrett

**Affiliations:** Enterprise Behavioral Health, Highmark Health, Pittsburgh, PA, USA; Department of Mental Health, Bloomberg School of Public Health, Johns Hopkins University, Baltimore, MD, USA; Enterprise Behavioral Health, Highmark Health, Pittsburgh, PA, USA; Enterprise Data & Analytics, Highmark Health, Pittsburgh, PA, USA; Enterprise Data & Analytics, Highmark Health, Pittsburgh, PA, USA; Enterprise Data & Analytics, Highmark Health, Pittsburgh, PA, USA

**Keywords:** insomnia, cognitive behavioral therapy, health care utilization and costs, psychiatric disorders

## Abstract

**Study Objectives:**

Insomnia has profound negative effects on behavioral health, physical health, and functional domains. Leveraging claims data from one of the nation’s largest payor-provider systems, the current study examined the real-world prevalence of insomnia, comorbidity of insomnia with behavioral health and other sleep disorders, and the impact of insomnia on total health care costs.

**Methods:**

Prevalence and costs associated with insomnia were assessed by examining claims data on approximately 3 million insured members during the year 2022. Using propensity score matching, total health care expenditures were calculated and compared for members with insomnia relative to matched cohorts without insomnia. Generalized linear modeling tested for differences between the cohorts.

**Results:**

Nine percent of members were identified as having insomnia; 64% of those also had depression, anxiety, and/or substance use disorder. Median total health care costs among individuals with insomnia were 4–6 times greater than among those without insomnia. A disproportionate amount (21.1%) of total claims spend came from members with insomnia.

**Conclusions:**

Findings demonstrate a high degree of clinical need and behavioral health comorbidity associated with insomnia within a large insured cohort. Beyond the clinical significance, the current results demonstrate substantial financial need and opportunity for adequately treating insomnia. This is especially the case for the high proportion of members with insomnia and cooccurring depression, anxiety, and/or substance use disorders. Overall, the findings point to the important role payors and providers may have in promoting greater attention to sleep and insomnia.

**Statement of Significance**

Insomnia has substantial negative effects on physical and behavioral health and overall functioning. The current article examined the prevalence and health care costs associated with insomnia in a sample of approximately 3 million insured members. Findings demonstrate high rates of insomnia among members, with the highest rates among older adults. Close to two-thirds of those with insomnia also had a depression, anxiety, and/or substance use disorder diagnosis. Median total health care costs among those with insomnia were 4-6 times greater than among those without insomnia. The findings point to the important role payors and providers may have in promoting greater attention to sleep and insomnia.

Insomnia is among the most pervasive, impactful, and treatable public health concerns in the United States. Approximately 10%–15% of adults experience an insomnia disorder and another 20% have subclinical insomnia symptoms [1, 2]. The rate of sleep disturbance in older adults has been reported to be as high as 50% [3, 4]. At the same time, untreated insomnia has been shown to have profound negative consequences in numerous domains [5, 6]. These include decreased quality of life [7–9], poor physical functioning [10], decreased ability to perform activities of daily living [7], increased risk of falls [11, 12], increased healthcare utilization [13], increased mortality and [14], increased risk of depression and other behavioral health conditions [7, 15, 16], In addition, a spate of research has demonstrated insomnia to be associated with increased risk of cardiovascular disease [17], cognitive decline [18–24], and Alzheimer’s disease [25–27]. In their review of the effects of insomnia, Sivertsen et al. [28] declared, “We conclude that insomnia predicts cumulative incidence of several physical and mental conditions.”

In addition to the clinical and functional impact of insomnia, available data in the literature point to the considerable costs of untreated insomnia and suggest significant financial savings associated with insomnia treatment, in terms of both health care costs (direct effects) and productivity, worker absenteeism, and quality of life (indirect effects) [29–34]. The costs associated with insomnia are particularly significant in older adults, largely due to increased fall and fracture risk associated with the use of hypnotics in this age group [35]. Moreover, left untreated, insomnia is often highly enduring in nature and further compounds negative financial impact [1]. While still developing in its empirical maturity, an accumulating body of research examining the impact of insomnia treatment on health care costs indicates that treatment of insomnia is associated with lower health care expenditures [31, 36, 37]. Consequently, the foregoing findings suggest that increased attention to and treatment of insomnia may confer numerous benefits central to the quintuple aim and reduced impact of care.

Unfortunately, insomnia is widely under-reported, unrecognized, and under-detected in primary care and other clinical settings due to numerous barriers at provider, systems, and patient levels [38, 39]. When insomnia is detected, it is frequently undertreated [40]. The mainstay treatment for insomnia consists of sleep medications, which have limited efficacy and can confer adverse reactions, especially when used for more than brief periods [41]. Although highly undetected and undertreated, insomnia is highly treatable. Cognitive Behavioral Therapy for Insomnia (CBT-I) is a highly efficacious, brief treatment recommended at the highest level and as first-line treatment for insomnia in clinical practice guidelines [42, 43]. Beyond its effectiveness in improving insomnia, CBT-I has also been shown to improve symptoms of depression and other behavioral health conditions that are often comorbid with insomnia [44, 45]. Yet, this gold-standard treatment remains widely unavailable in real-world treatment settings [46].

The current study was conducted to examine the prevalence of insomnia, as well as cooccurring behavioral health conditions, and the costs associated with members with insomnia for informing decisions and plans for structured implementation of CBT-I within one of the nation’s largest payor-provider health systems [47]. Leveraging a robust claims data system encompassing nearly 3 million members nationally, the current evaluation examined (1) the current prevalence of insomnia and cooccurring behavioral health conditions and (2) the health care costs associated with individuals with insomnia and cooccurring behavioral health conditions among insured members across multiple insurance product lines (Commercial, Affordable Care Act, Medicare Advantage, and Medicaid). Specific questions the evaluation seeks to address include: what is the prevalence of insomnia and cooccurring behavioral health conditions in a large real-world community sample of insured members? What are the total health care costs associated with members with insomnia? What are the incremental and relative health care costs associated with having different types of cooccurring behavioral health conditions with insomnia? It was anticipated that, relative to individuals without insomnia, the total health care expenditures of members with insomnia would be significantly higher—and even more so among those with insomnia and cooccurring behavioral health needs (i.e. depression, anxiety, and/or substance use disorder).

## Method

### Data extraction and identification

Insomnia prevalence and expenditure analyses were conducted on all approved claims from January 01, 2022 through December 31, 2022, using all adult members between 18 and 100 years old with at least 6 months of continuous enrollment during 2022 (*N* = 2.9 million members). Members were identified as having insomnia if any of the following criteria were met during the 2022 calendar year: a claim with an insomnia diagnosis in any diagnosis code field; a claim with an Episode Treatment Group (ETG) code indicating insomnia; or a claim for insomnia-related medication, consistent with past approaches [48, 49]. We also reviewed available electronic health record (EHR) data for encounters containing a diagnosis of insomnia and cross-referenced the corresponding patients within the claims database to be fully inclusive in the identification of members with insomnia. Insomnia-related medications included benzodiazepines FDA-approved for the treatment of insomnia, nonbenzodiazepine sedative-hypnotics (i.e. Z-drugs), orexin receptor antagonists, melatonin receptor agonists, select antidepressants prescribed at low doses for sleep (e.g. doxepin, trazodone), and select first-generation antihistamines commonly used to treat insomnia (e.g. doxylamine succinate). See Supplementary Table S1 in the Appendix for the full list of medications.

### Prevalence analyses

Prevalence estimates were calculated by taking the count of members identified with insomnia and dividing it by the total count of all included members. Subgroup analyses were then conducted to stratify by sex, age, and insurance type. In addition to the examination of the prevalence of insomnia, we examined the prevalence of one or more core behavioral health conditions (depression, anxiety, and/or substance use disorder) using ETG codes and ICD-10 codes from claims, as well as non-insomnia sleep conditions, consistent with the International Classification of Sleep Disorders—Third Edition (ICSD-3-TR). Non-insomnia sleep disorders included (1) sleep-related breathing disorders (e.g. obstructive sleep apnea, central sleep apnea syndromes), (2) central disorders of hypersomnolence (e.g. narcolepsy, idiopathic hypersomnia), (3) circadian rhythm sleep–wake disorders (e.g. delayed sleep–wake phase disorder), (4) parasomnias (REM-related parasomnias; e.g. nightmare disorder and NREM-related parasomnias; e.g. sleepwalking), and (5) sleep-related movement disorders (e.g. restless leg syndrome, sleep-related bruxism).

### Expenditure analyses

Expenditure analyses examined the total health care costs of members with insomnia relative to a matched group of members without insomnia. First, the proportion of total health care expenditures in 2022 attributable to members with insomnia was calculated. Specifically, we determined the average TCC by calculating the per member per month (PMPM) spend for members with insomnia. PMPM spending was calculated using the sum of the allowed amounts across all claims for each member divided by the number of months that member was enrolled during 2022. To account for other factors that may impact costs, we compared PMPM spending between members with insomnia to a matched cohort of members without insomnia. Next, to examine the financial impact of having insomnia while having cooccurring behavioral health conditions, we compared the costs of those with insomnia and comorbid depression, anxiety, and/or substance use disorder to the costs of matched cohorts without insomnia but with the same comorbid behavioral health condition(s). In total, there were five matched cohorts created for cost comparison—a broad cohort of those with and without insomnia (regardless of comorbidities), and four on the basis of the comorbidity groupings. The five cohort comparisons are listed below:

1. Insomnia versus no insomnia.
2. Insomnia + depression versus no insomnia + depression.
3. Insomnia + anxiety versus no insomnia + anxiety.
4. Insomnia + substance use disorder versus no insomnia + substance use disorder.
5. Insomnia + 1 or more of these comorbidities versus no insomnia + 1 or more of these comorbidities.

The matched no-insomnia groups for the five cohort-based comparisons were created using propensity score matching for the average treatment effect on the treated (i.e. the estimated impact of insomnia based on those in the sample who were identified as having insomnia). Matching was performed with replacement, such that a comparison member without insomnia could serve as the matched counterpart for more than one member with insomnia. Propensity scores were estimated using a logistic model; the predictors in the model—the characteristics the groups were matched on—were demographic characteristics and comorbidities (e.g. age, sex, depression, anxiety, all comorbidities in the Charlson Comorbidity Index) [50]. Exact matching was used for sex. The adequacy of the matching (i.e. the balance of the groups) was assessed using standardized mean differences, variance ratios, Kolmogorov-Smirnov statistics (“empirical cumulative density functions”), and screening for outliers. With the matched samples, PMPM spending was compared using descriptive statistics—both mean and median to provide representative estimates of the typical individual PMPM—and generalized linear regression modeling. The regression models used a gamma distribution and a natural logarithm link. Prevalence and expenditure analyses were conducted using RStudio Workbench (v 1.4.1717-3) running *R* (v. 4.0.0).

## Results

Rates of insomnia and core behavioral health comorbidities across all members and by specific insured segments are reported in Table 1. As Table 1 reveals, the overall frequency of insomnia across adult members in the year 2022 with 6 or more months of continuous enrollment was 8.9%. Of the members with insomnia, a majority (64%) also had a depression, anxiety, and/or substance use disorder diagnosis. Among members with depression, anxiety, and/or substance use disorders, 20.1% had an insomnia diagnosis. Prevalence of insomnia varied by member characteristics, with higher rates observed for females, older individuals, and Medicare Advantage members. Of those with insomnia, 28.0% also had a sleep-related breathing disorder, 7.9% had a sleep-related movement disorder, 6.6% had hypersomnolence, 1.7% had a diagnosis of circadian rhythm sleep disorders, and 1.5% had parasomnia. Across the full membership, a disproportionate amount (21.1%) of all expenditures were attributable to members with insomnia.

**Table 1.** Percentages of Insured Members (With 6 Months of Continuous Enrollment) With Insomnia, Combinations of Insomnia and Behavioral Health Comorbidities, and Corresponding Demographic Characteristics of the Aforementioned Groupings

	Population							
Diagnoses	Overall	Male	Female	Age < 65	Age ≥ 65	Medicare advantage	Private insurance	Other insurance
*Of all members (N = 2 824 550)*								
Insomnia (*n* = 252 233)	8.93%	7.40%	10.22%	7.86%	13.09%	17.28%	7.84%	9.02%
Insomnia and depression (*n* = 92 793)	3.29%	2.15%	4.24%	2.91%	4.73%	7.20%	2.84%	3.09%
Insomnia and anxiety (*n* = 129 777)	4.59%	3.16%	5.81%	4.28%	5.82%	8.49%	4.20%	4.24%
Insomnia and SUD (*n* = 25 638)	0.91%	0.96%	0.87%	0.92%	0.84%	1.51%	0.89%	0.71%
Insomnia and 1 + COM (*n* = 161 414)	5.71%	4.17%	7.02%	5.21%	7.69%	11.19%	5.13%	5.32%
No insomnia and 1 + COM (*n* = 641 327)	22.71%	17.29%	27.26%	23.29%	20.43%	23.08%	23.22%	20.76%
*Of members with insomnia (n = 252 233)*								
1 + COM (*n* = 161 414)	63.99%	56.29%	68.70%	66.24%	58.74%	64.76%	65.44%	58.99%
*Of members with 1+ COM (n = 802 741)*								
Insomnia (*n* = 161 414)	20.11%	19.42%	20.47%	18.28%	27.35%	32.65%	18.10%	20.39%

SUD, substance use disorder; COM, comorbidity. Comorbidities included depression, anxiety, and SUD.

The propensity score matching for the five cohorts was adequately balanced across all demographic characteristics and comorbidities. That is, all balance metrics were within generally acceptable limits. For instance, all absolute standardized mean differences were less than 0.2, variance ratios were all less than 2.0, and Kolmogorov-Smirnov statistics (“empirical cumulative density function maximum”) were all below 0.11. The sample sizes of the matched cohorts are reported in Table 2. The results of the expenditure analyses comparing those with insomnia to matched groups without insomnia reveal that individuals with insomnia have 2 to 3 times higher mean total care costs and 4 to 6 times higher median total care costs than their non-insomnia counterparts, with expenditures generally even greater in the presence of behavioral health comorbidity (Table 2).

**Table 2.** Mean, Median, Mean Ratio, and Median Ratio Showing Per-member Per-month Cost Differences in Each Matched Cohort

Comparison	Mean	Median	Mean ratio	Median ratio
Insomnia vs. no insomnia			2.10	4.45
No insomnia (*n* = 159 850)	$867	$116		
Insomnia (*n* = 252 233)	$1823	$516		
Insomnia and depression vs. no insomnia and depression			2.97	5.72
No insomnia and depression (*n* = 40 421)	$725	$127		
Insomnia and depression (*n* = 92 793)	$2150	$727		
Insomnia and anxiety vs. no insomnia and anxiety			2.57	5.09
No insomnia and anxiety (*n* = 61 768)	$821	$127		
Insomnia and anxiety (*n* = 129 777)	$2107	$646		
Insomnia and SUD vs. No insomnia and SUD			2.35	4.01
No insomnia and SUD (*n* = 13 421)	$1430	$314		
Insomnia and SUD (*n* = 25 638)	$3355	$1260		
Insomnia and at least one comorbidity vs. no insomnia and at least one comorbidity			2.59	4.55
No insomnia and at least one comorbidity (*n* = 80 673)	$798	$140		
Insomnia and at least one comorbidity (*n* = 161 414)	$2066	$637		

SUD, substance use disorder. “At least one comorbidity” refers to at least one of depression, anxiety, and/or SUD.

To further explore these descriptive findings, generalized linear regression models were used to compare the total cost of care (TCC) for those with insomnia and their matched counterparts without insomnia. Using a natural logarithm link, the models naturally accounted for the skewed distribution of the costs and provided estimates that approximate percent differences in costs. Results of these models are displayed in Table 3. Consistently, those with insomnia had significantly higher costs (all *p* < .001); members with insomnia, with no consideration of comorbid conditions, were estimated to have 74.1% (95% CI:[72.3%, 76.1%]) higher costs than those without insomnia. That is, regardless of the spending for a member without insomnia, a similar member with insomnia would be predicted to have 75% higher costs. All comorbid comparisons showed even higher estimated differences, ranging from 85% higher (substance use disorders) to over 108% higher costs (depression).

**Table 3.** Generalized Linear Regression Models Comparing Total Cost of Care for Members With and Without Insomnia in Each Matched Cohort

Comparison	Estimate	95% CI	*P*-value	Observations
Insomnia vs. no insomnia	0.742	0.723, 0.761	<.001	533 422
Insomnia and depression vs. no insomnia and depression	1.086	1.058, 1.114	<.001	195 424
Insomnia and anxiety vs. no insomnia and anxiety	0.941	0.916, 0.966	<.001	272 723
Insomnia and SUD vs. no insomnia and SUD	0.853	0.800, 0.906	<.001	54 112
Insomnia and at least one comorbidity vs. no insomnia and at least one comorbidity	0.949	0.928, 0.971	<.001	340 410

All models were specified with a gamma distribution and natural logarithm link.

## Discussion

Although widely under-recognized and under-treated, insomnia has among the greatest clinical, functional, and economic costs on patients, providers, and payors. To help shed light on the clinical and financial need and opportunity for addressing insomnia, the current study examined the current prevalence of insomnia and comorbid behavioral health conditions, as well as costs associated with members with such conditions.

The results with respect to prevalence reveal a high rate of insomnia, especially so among older members. Furthermore, it is noteworthy that close to two-thirds of those with insomnia also had a depression, anxiety, and/or substance use disorder diagnosis. These findings are consistent with previous research showing cooccurrence of insomnia with depression and anxiety in particular, a relationship that is likely bidirectional [51], although the findings of the current analysis, which leverage claims data and extend beyond a treatment-seeking patient population, suggest that the cooccurrence may be even greater than previously recognized [52, 53].

The results of the analyses examining health care costs associated with individuals with insomnia add to the early body of literature showing the significant financial impact of insomnia and suggest a large financial opportunity associated with addressing insomnia [30, 32, 54, 55]. It is noteworthy that median total health care costs among individuals with insomnia were 4–6 times greater than among those without insomnia, and mean total costs were 2–3 times greater. Furthermore, 21% of total claim spend came from members with insomnia; while these costs cannot be attributed directly to insomnia, this amount exceeds what would be expected based on the proportional representation of insomnia (8.9%) in the total membership.

While direct comparison of the results from the financial impact analyses with those from prior studies is difficult due to differences in design, sample, and methods in studies of this type, there are some notable observations when comparing the current findings with past analyses. In some instances, expenditures associated with insomnia documented in the current evaluation are higher than those reported previously. For example, in their comparison of claims data for patients with and without insomnia, Ozminkowski and colleagues found that those with insomnia had mean TCC approximately 1.25 times higher than those without insomnia over a 6-month period, and this ratio was comparable for older (≥65) and younger adults (18–64) [30]. Furthermore, in comparisons of 12 months of claims data for patients with a depressive disorder with and without insomnia, Asche and colleagues found that older adults (≥65) with cooccurring insomnia had mean TCC 1.17 times higher than those with a depressive disorder without insomnia, and younger adults (18–64) had total costs 1.21 times higher [54]. Notably, the differences may be related in significant part to differences in how insomnia was operationalized and in the specific samples (e.g. mixed-age, older adults, etc.) across studies. For instance, relative to Ozminkowski and colleagues, the current study was more inclusive in terms of insomnia diagnoses and insomnia-related medications. Relative to Asche and colleagues, the current study was more inclusive in terms of medications to identify insomnia. Whereas Asche and colleagues excluded antidepressant medications that could be used to treat insomnia or depression, we specified dose requirements for these medications (e.g. trazodone, doxepin, and amitriptyline) consistent with their use in the treatment of insomnia, potentially optimizing sensitivity to detect insomnia as well as specificity to identify cases as insomnia versus depression. Furthermore, the current study was more specific and conservative in its identification of insomnia compared to Asche and colleagues, who included sleep disorders other than insomnia in their definition (e.g. hypersomnia unspecified).

Other studies have reported higher direct expenditures associated with insomnia, though included additional costs than those accounted for in the current analyses and used methods other than propensity matching. For example, Daley and colleagues, using self-report data from a large Canadian sample, included costs associated with alcohol used as a sleep aid [55]. Wickwire and colleagues analyzed claims data for a sample of older Medicare beneficiaries to compare patients with and without insomnia but used statistical methods to control for covariates instead of propensity matching and included the cost of nursing home care [32]. Notably, the findings from the current regression analyses demonstrating the TCC for those with insomnia to be estimated to be 74% higher than those without insomnia is in line with the results from smaller analyses of health plan members showing that those with moderate and severe insomnia had total health care costs that were, on average, 75% larger than those without insomnia [34].

The high prevalence and considerable costs associated with insomnia point to the significant need and opportunity to promote the availability of gold-standard treatment for insomnia. In fact, the current findings served as a key basis for the development of a structured implementation initiative to establish new capability to deliver CBT-I within routine primary care and behavioral health settings of the Allegheny Health Network (see article reporting on patient and provider outcomes from this pilot implementation initiative in this issue) [47].

While the current evaluation has a number of strengths, there are important limitations that should be considered. With respect to the analysis of health care costs of members with and without insomnia, it is important to highlight that while insomnia may contribute to increased health care costs, the current analysis is descriptive in nature and does not provide a causal link demonstrating that the increased cost is directly related to insomnia. However, a strength of the current analysis that supports these differences as likely due to insomnia was the use of matched comparisons of members with and without insomnia, which reduces the potential influence of other variables in the analysis of costs associated with insomnia.

Two additional limitations are worth noting. First, in the prevalence analyses, it was not possible to stratify prevalence by race/ethnicity, as this information is often not included in claims and therefore not reliably available. Second, in assuming a more conservative approach, consistent with other recent approaches [32], we excluded some medications (e.g. low-dose quetiapine) that may be prescribed for sleep problems given that these medications may be used for other purposes (e.g. initiation of treatment for psychosis, augmentation for depression treatment). Accordingly, the current findings related to prevalence of insomnia may under-reflect the true rate of insomnia in the community.

In sum, the foregoing results related to the prevalence and financial impact of insomnia suggest that greater attention be paid to addressing insomnia and its common cooccurrence and relationship with other conditions. The findings reported herein suggest significant clinical and financial impact opportunity to payors for promoting identification of insomnia and increasing the availability of evidence-based treatment. Given the limited recognition, under-reporting, and under-detection of insomnia that is pervasive within primary care and specialty settings [38, 39], the current study suggests the potential opportunity for payors and providers to leverage indicators (e.g. medications, diagnostic information) within claims and EHR data, such as those methods used within this study, to systematically identify possible insomnia and, when identified, proactively outreach to members and/or PCPs for further assessment and referral to treatment. Such a proactive identification and outreach approach offers considerable potential for greatly increasing and accelerating detection and for promoting engagement in effective treatment.

## Supplementary Material

Supplementary material is available at *SLEEP Advances* online.

zpae054_suppl_Supplementary_Material
