# Supplementary material for: Clinical and financial significance of insomnia within a large payor-provider health system
Source: Sleep Adv. 2024 Jul 27;5(1):zpae054. doi: 10.1093/sleepadvances/zpae054 (PMC11450625; doi:10.1093/sleepadvances/zpae054)
Supplement: zpae054_suppl_Supplementary_Material [file zpae054_suppl_supplementary_material.docx]

Clinical and Financial Significance of Insomnia within a Large Payor-Provider Health System

Supplemental Material

Bradley E. Karlin, PhD, ABPP, MBA^1,2^

Ryan J. Anderson, PhD^1^

Jillian M. Rung, PhD^3^

Charlotte Drury-Gworek, MSN, RN^3^

Tyson S. Barrett, PhD^3^

^1^Enterprise Behavioral Health, Highmark Health, Pittsburgh, PA, USA

^2^Department of Mental Health, Bloomberg School of Public Health, Johns Hopkins University, Baltimore, MD, USA

^3^Enterprise Data & Analytics, Highmark Health, Pittsburgh, PA, USA

Authors’ Note: This project was supported by the Richard King Mellon Foundation (Grant #10714). Correspondence concerning this article should be addressed to Dr. Bradley E. Karlin, bradkarlin@gmail.com

**Supplemental Material**

Table S1. List of all medications used for identification of insomnia.

| Ambien | Diazepam 2 Mg Tablet | Mirtazapine 7 5 Mg Tablet | Temazepam Cap 15 Mg |
| --- | --- | --- | --- |
| Ambien Cr | Diazepam 5 Mg 5 Ml Solution | Mirtazapine Tab 15 Mg | Temazepam Cap 22 5 Mg |
| Ambien Cr 12 5 Mg Tablet | Diazepam 5 Mg Ml Oral Conc | Mirtazapine Tab 15 Mg Odt | Temazepam Cap 30 Mg |
| Ambien Cr Tab 12 5 Mg | Diazepam 5 Mg Tablet | Mirtazapine Tab 7 5 Mg | Temazepam Cap 7 5 Mg |
| Ambien Cr Tab 6 25 Mg | Diazepam Tab 10 Mg | Multi Ingredient Compound | Trazodone |
| Ambien Tab 10 Mg | Diazepam Tab 2 Mg | Multi Ingredient Compound Barbital | Trazodone 100 Mg Tablet |
| Amitriptyline Hcl | Diazepam Tab 5 Mg | Pentobarbital | Trazodone 150 Mg Tablet |
| Amitriptyline Hcl 10 Mg Tab | Diphenhydramine | Perphenazine Amitriptyline | Trazodone 50 Mg Tablet |
| Amitriptyline Hcl 25 Mg Tab | Doral | Phenobarb | Trazodone Hcl |
| Amitriptyline Hcl 50 Mg Tab | Doxepin | Phenobarb Elx 20 Mg 5 Ml | Trazodone Tab 100 Mg |
| Amobarbital | Doxepin Hcl | Phenobarb Sol 20 Mg 5 Ml | Trazodone Tab 150 Mg |
| Ativan | Doxepin Tab 3 Mg | Phenobarb Tab 100 Mg | Trazodone Tab 50 Mg |
| Belladonna Phenobarbital | Doxepin Tab 6 Mg | Phenobarb Tab 15 Mg | Triazolam |
| Belsomra | Doxylamine | Phenobarb Tab 16 2 Mg | Triazolam 0 125 Mg Tablet |
| Belsomra 10 Mg Tablet | Edluar | Phenobarb Tab 30 Mg | Triazolam 0 25 Mg Tablet |
| Belsomra Tab 10 Mg | Edluar 10 Mg Sl Tablet | Phenobarb Tab 32 4 Mg | Triazolam 100 Mg |
| Belsomra Tab 15 Mg | Edluar Sub 10 Mg | Phenobarb Tab 60 Mg | Triazolam Tab 0 125 Mg |
| Belsomra Tab 20 Mg | Enovarx Amitriptyline | Phenobarb Tab 64 8 Mg | Triazolam Tab 0 25 Mg |
| Belsomra Tab 5 Mg | Estazolam | Phenobarb Tab 97 2 Mg | Unisom Tab 25 Mg |
| Clonazep Odt Tab 0 125 Mg | Estazolam Tab 2 Mg | Phenobarbital | Valium |
| Clonazep Odt Tab 0 25 Mg | Eszopiclone | Phenobarbital 100 Mg | Valium Tab 5 Mg |
| Clonazep Odt Tab 0 5 Mg | Eszopiclone 1 Mg Tablet | Phenobarbital 30 Mg Tablet | Valtoco Spr 15 Mg |
| Clonazep Odt Tab 1 Mg | Eszopiclone 100 Mg | Phenobarbital 32 4 Mg Tablet | Valtoco Spr 20 Mg |
| Clonazep Odt Tab 2 Mg | Eszopiclone 2 Mg Tablet | Phenobarbital 64 8 Mg Tablet | Xyrem |
| Clonazepam | Eszopiclone 3 Mg Tablet | Phenobarbital 97 2 Mg Tablet | Xywav |
| Clonazepam 0 125 Mg Dis Tab | Eszopiclone Tab 1 Mg | Phenobarbital Belladon | Zaleplon |
| Clonazepam 0 25 Mg Odt | Eszopiclone Tab 2 Mg | Phenobarbital Belladonna | Zaleplon 10 Mg Capsule |
| Clonazepam 0 5 Mg Dis Tablet | Eszopiclone Tab 3 Mg | Phenobarbital Hyosc at | Zaleplon 100 Mg |
| Clonazepam 0 5 Mg Tablet | Famotidine | Phenobarbital Sodium | Zaleplon 5 Mg Capsule |
| Clonazepam 1 Mg Dis Tablet | Flurazepam 15 Mg Capsule | Prudoxin | Zaleplon Cap 10 Mg |
| Clonazepam 1 Mg Tablet | Flurazepam Cap 15 Mg | Quazepam | Zaleplon Cap 5 Mg |
| Clonazepam 2 Mg Tablet | Flurazepam Cap 30 Mg | Ra Sleep Aid Tab 25 Mg | Zolpidem |
| Clonazepam 60 Mg | Halcion | Ramelteon | Zolpidem 100 Mg |
| Clonazepam Tab 0 5 Mg | Halcion Tab 0 25 Mg | Ramelteon Tab 8 Mg | Zolpidem 100 Mg |
| Clonazepam Tab 1 Mg | Hydrocortisone | Remeron | Zolpidem Er Tab 12 5 Mg |
| Clonazepam Tab 2 Mg | Intermezzo | Restoril | Zolpidem Er Tab 6 25 Mg |
| Cresol | Intermezzo Sub 3 5 Mg | Rozerem | Zolpidem Tab 10 Mg |
| Dayvigo | Klonopin | Rozerem 8 Mg Tablet | Zolpidem Tab 5 Mg |
| Dayvigo Tab 10 Mg | Klonopin 0 5 Mg Tablet | Rozerem Tab 8 Mg | Zolpidem Tar Sub 1 75 Mg |
| Dayvigo Tab 5 Mg | Klonopin 1 Mg Tablet | Seconal Sodium | Zolpidem Tar Sub 3 5 Mg |
| Dexmedetomidine | Lorazepam | Silenor | Zolpidem Tart Er 12 5 Mg Tab |
| Dexmedetomidine Hcl | Lorazepam Inj 2 Mg Ml | Silenor 6 Mg Tablet | Zolpidem Tart Er 6 25 Mg Tab |
| Diastat | Lunesta | Silenor Tab 6 Mg | Zolpidem Tartrate |
| Diastat Acdl Gel 5 10 Mg | Lunesta 3 Mg Tablet | Sleep Aid Tab 25 Mg | Zolpidem Tartrate 10 Mg Tablet |
| Diastat Acudial | Midazolam | Sleep Tab 25 Mg | Zolpidem Tartrate 5 Mg Tablet |
| Diastat Acudial 5 7 5 10 Mg Kt | Midazolam Hcl | Temazepam | Zolpidem Tartrate Er |
| Diastat Ped Gel 2 5 m Gel | Midazolam Syp 2 Mg Ml | Temazepam 15 Mg Capsule | Zonalon |
| Diazapam 100 | Mirtazapine | Temazepam 22 5 Mg Capsule |  |
| Diazepam | Mirtazapine 15 Mg Odt | Temazepam 30 Mg Capsule |  |
| Diazepam 10 Mg Tablet | Mirtazapine 15 Mg Tablet | Temazepam 7 5 Mg Capsule |  |
